# Improved Protein Identification in Shotgun Proteomics with a Group-Level Extension of the LPGF Model

**DOI:** 10.1021/acs.jproteome.5c01253

**Published:** 2026-07-20

**Authors:** Gorka Prieto, Jesús Vázquez

**Affiliations:** † Department of Communications Engineering, University of the Basque Country (UPV/EHU), 48013 Bilbao, Spain; ‡ Cardiovascular Proteomics Lab, 38799 Centro Nacional de Investigaciones Cardiovasculares Carlos III (CNIC), 28029 Madrid, Spain; § CIBER de Enfermedades Cardiovasculares (CIBERCV), 28029 Madrid, Spain

**Keywords:** FDR, proteomics, protein identification, target-decoy approach

## Abstract

Shotgun proteomics relies on robust statistical methods to increase the confidence of the protein identifications. Previously, we introduced LPGF, a protein probability model based on unique peptides. In this study, we extend LPGF to protein groups that share peptides by considering only peptides that are unique to each group. This strategy preserves the principle of unique evidence while enabling the probability estimation at the protein-group level. Applying our method to three tissues from the Human Proteome Map (HPM), we evaluated the gain in protein identifications obtained with group-unique peptides compared to those obtained using only protein-unique peptides and different scores. The accuracy of false discovery rate (FDR) estimation was further validated using a standard data set for protein inference based on human *Protein Epitope Signature Tags* (PrESTs), as well as through an entrapment experiment using the substantially larger HPM data set. To facilitate adoption, we developed an R package (b10prot) that integrates the full workflow, including protein-level and group-level LPGF score computation and protein grouping via an R port of our previous tool PAnalyzer. Our results demonstrate that extending LPGF to protein groups increases sensitivity while maintaining robust FDR control, thus providing the proteomics community with a practical framework for more comprehensive protein identification.

## Graphical abstract

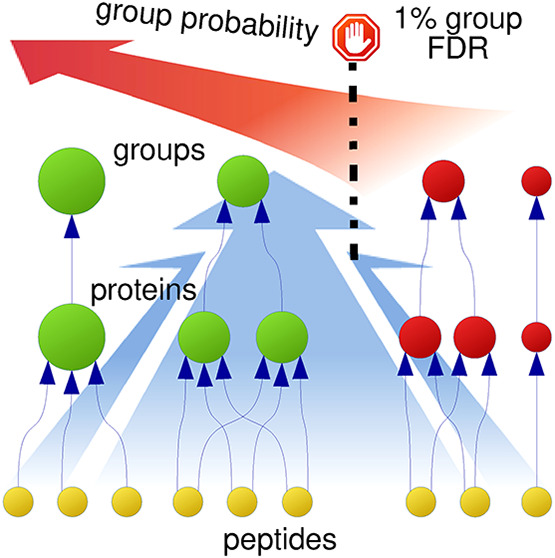

## Introduction

Shotgun proteomics using tandem mass spectrometry (MS/MS) has become a central approach to large-scale protein identification in complex biological samples. Although peptide-spectrum matching has substantially improved in accuracy and throughput, several major challenges remain at the protein level. These include not only the reconstruction of proteins from peptide evidence (protein inference) but also accurate control of false discovery rate (FDR) at the protein level.

In protein inference, a central difficulty is that identified peptides often map to more than one protein sequence, creating ambiguity in the reconstruction of the underlying proteome.^1,2^ Shared peptides are common in eukaryotic proteomes due to homologous gene families, alternative splicing, and incomplete proteotypic coverage, and their presence complicates both protein identification and quantification. Some approaches circumvent the ambiguity introduced by shared peptides by only considering peptides that uniquely map to a single protein. Although this strategy reduces complexity, it inevitably discards proteins supported exclusively by shared evidence and may underestimate the confidence of proteins with a mixture of unique and shared peptides.

Protein grouping has become an essential strategy for handling shared peptides in shotgun proteomics. Instead of attempting to assign each peptide to a single protein, grouping algorithms cluster proteins into sets that are indistinguishable on the basis of observed peptide evidence. These approaches preserve all identified peptides, including shared ones, providing a coherent representation of ambiguity through the grouping rules. As a result, protein groups have emerged as the standard reporting unit in many proteomics pipelines,^3^ offering a principled compromise between discarding shared peptides and overinterpreting ambiguous evidence.

Beyond inference ambiguity, protein-level error control is affected by the propagation of errors across the hierarchical levels. It is well-known that the false discovery rate (FDR) typically increases when moving from peptide- to protein-level identifications, even when peptide-level FDR is accurately controlled.^4^ Inspection of the algorithms employed by commonly used software tools^5^ revealed that, independent of the method used to derive peptide probabilities, the majority of them use either information from the best peptide of each protein or simple multiplicative operations to integrate peptide probabilities into protein probability. In a previous work, we proposed improved versions of the best-peptide method (LPGM) and introduced LPGF,^5^ a probabilistic model for protein identification that computes protein-level probabilities using all available information from unique peptides, including both the probabilities of identified peptides and the number of nonidentified peptides associated with each protein. LPGF provided a more accurate estimation of decoy protein probabilities compared to methods that rely on the best peptide (LPGM and LPM) or on identified peptides alone (LPF).

However, a key limitation of the original LPGF formulation was that it could only be applied to proteins with at least one unique peptide, effectively excluding proteins represented solely by shared sequences. In our previous study, we partially mitigated this ambiguity by applying LPGF at the gene level rather than working strictly at the protein level. In the present work, we further address this limitation by extending LPGF to both protein-group and gene-group levels, reporting results for both, although we often refer to protein groups for simplicity, or simply to groups. To implement this extension, we redesigned our previously developed protein-group method, PAnalyzer^6^, to make it compatible with LPGF. More precisely, we introduced a two-step procedure to arrange relationships between peptides, proteins, and groups, taking into account peptides that did not pass the peptide identification threshold but that were needed for computing LPGF. We define and exploit peptides that are exclusive to each group (“group-unique” peptides), and redefine the LPGF framework to be applied to all groups produced by the protein-grouping algorithm. Finally, we show that the group-level probabilities calculated by LPGF are well-calibrated, and use FDR-testing methods to demonstrate that our previously proposed FDRn and FDRr estimates provide accurate control of FDR at the group level.

We evaluated the method using three tissues from the Human Proteome Map (HPM)^7^ to assess the identification performance. We then validated group-level FDR estimation using a standard protein inference benchmark based on human *Protein Epitope Signature Tags* (PrESTs),^8^ as well as through an entrapment experiment using the HPM data set to reflect data set sizes commonly encountered in practice.

To facilitate adoption by the community, we provide an open-source R package, named b10prot, which integrates the complete workflow, including PAnalyzer-based protein grouping and computation of protein- and group-level LPGF scores. The package is freely available at https://akrogp.github.io/b10prot.

## Experimental Section

### HPM Data Set

For the protein identification performance study, we selected the same data set that we used in our previous study,^5^ consisting of one of the first drafts of the human proteome, submitted to ProteomeXchange^9^ as PXD000561.^7^ This data set is known as the Human Proteome Map (HPM) and contains experimental data from different human tissues. We carried out comparisons at this tissue level and validated the reproducibility of the results in three tissues: Adult_Heart, Adult_Liver, and Adult_Testis. We selected these tissues because they span the data set size range of the HPM: Adult_Heart has a lower number of identifications, Adult_Testis has one of the largest numbers of identifications, and Adult_Liver is an intermediate case. Raw files for each tissue were converted to MGF file format using ProteoWizard^10^ with the --filter ‘‘peakPicking true 1-’’ option of msconvert.

As the target database, we used UniProt release 2026_01, including isoform sequences to evaluate the effect of grouping not only at the gene level but also at the protein level. Common contaminants were downloaded from the Common Repository of Adventitious Proteins (cRAP), collated by the Global Proteome Machine (GPM) organization (ftp://ftp.thegpm.org/fasta/cRAP/crap.fasta). These sequences were concatenated to the target database after adding a CRAP_ prefix and converting line endings using the dos2unix utility. Finally, isoleucine residues were replaced with leucine residues using a custom Python script.

Database searches were performed using the Crux toolkit^11,12^ (version 4.3.2). Peptide indexing was carried out with tide-index, and spectra were searched using tide-search. For each tissue, individual MGF files were searched separately, and the resulting search outputs were subsequently combined into a single file per tissue. This combined file was then processed with Percolator^13,14^ to obtain peptide-level confidence estimates. The following parameters were used to generate the search index: trypsin specificity with up to two missed-cleavages, a minimum peptide length of 6 amino acids, and reversed peptide sequences for decoy generation (decoy-format = peptide-reverse) using a concatenated target-decoy strategy (concat = T). Spectral searches were performed using a precursor mass tolerance of 10 ppm (precursor-window = 10, precursor-window-type = ppm), with fragment ion binning parameters mz-bin-width = 0.02 and mz-bin-offset = 0.0. Carbamidomethylation of cysteine residues was specified as a static modification (+57.02146 Da), and oxidation of methionine was included as a variable modification (+15.9949 Da) with a maximum of two variable modifications per peptide (mods-spec = C+57.02146,2M+15.9949).

An entrapment data set was also generated from the target database described above (including isoforms, contaminants, and isoleucine substitutions) using the FDRBench application^15^ (version 0.0.4) with the following parameters: -level protein, -uniprot, -fix_nc c, and -check. The resulting entrapment database was subsequently used for database searching by using the same search workflow described above.

### PrEST Data Set

For validating the protein identifications, we used the PrEST gold-standard data set.^8^ To avoid differences with the original results^8^ due to previous data processing steps, we replicated all of the processing steps in the original study to obtain the complete peptide-level results that were used as input to our identification pipeline. More precisely, raw data files were downloaded from ProteomeXchange^9^ PXD008425, converted to MS1 and MS2 formats using ProteoWizard^10^ with the --filter ‘‘peakPicking true 2-’’ option of msconvert and subsequently processed by Hardklor^16^ followed by Bullseye,^17^ through the interface of the Crux 2.1 package,^11^ to set monoisotopic masses. The resulting MS2 spectra were then matched to separate target and decoy sequence databases using Tide within Crux 2.1 ^11,12^ with no variable modifications, 10 ppm search tolerance, protein-reverse decoy format, 2 missed-cleavages, and all other parameters kept at their default values. Finally, Percolator v3.01 ^13,14^ was used to derive peptide-level probabilities from the different mass spectrometry runs.

### Algorithms and Tools

PAnalyzer^6^ is a protein inference tool developed for shotgun proteomics that systematically classifies identified peptides and groups proteins according to their peptide evidence. Peptides are first categorized into three types: unique peptides, which are assigned to a single protein; discriminating peptides, which are shared among proteins without unique peptides; and non-discriminating peptides, which are shared but explained by proteins that have unique or discriminating peptides. On the basis of these peptide types, proteins are classified into four evidence categories: conclusive proteins (having at least one unique peptide), non-conclusive proteins (containing only non-discriminating peptides), indistinguishable proteins (sharing the same discriminating peptides), and ambiguous groups (groups of proteins that jointly explain the presence of discriminating peptides). This classification, including an additional category of non-significant peptides that are not used for grouping, is illustrated in Figure 1.

**Figure 1:**
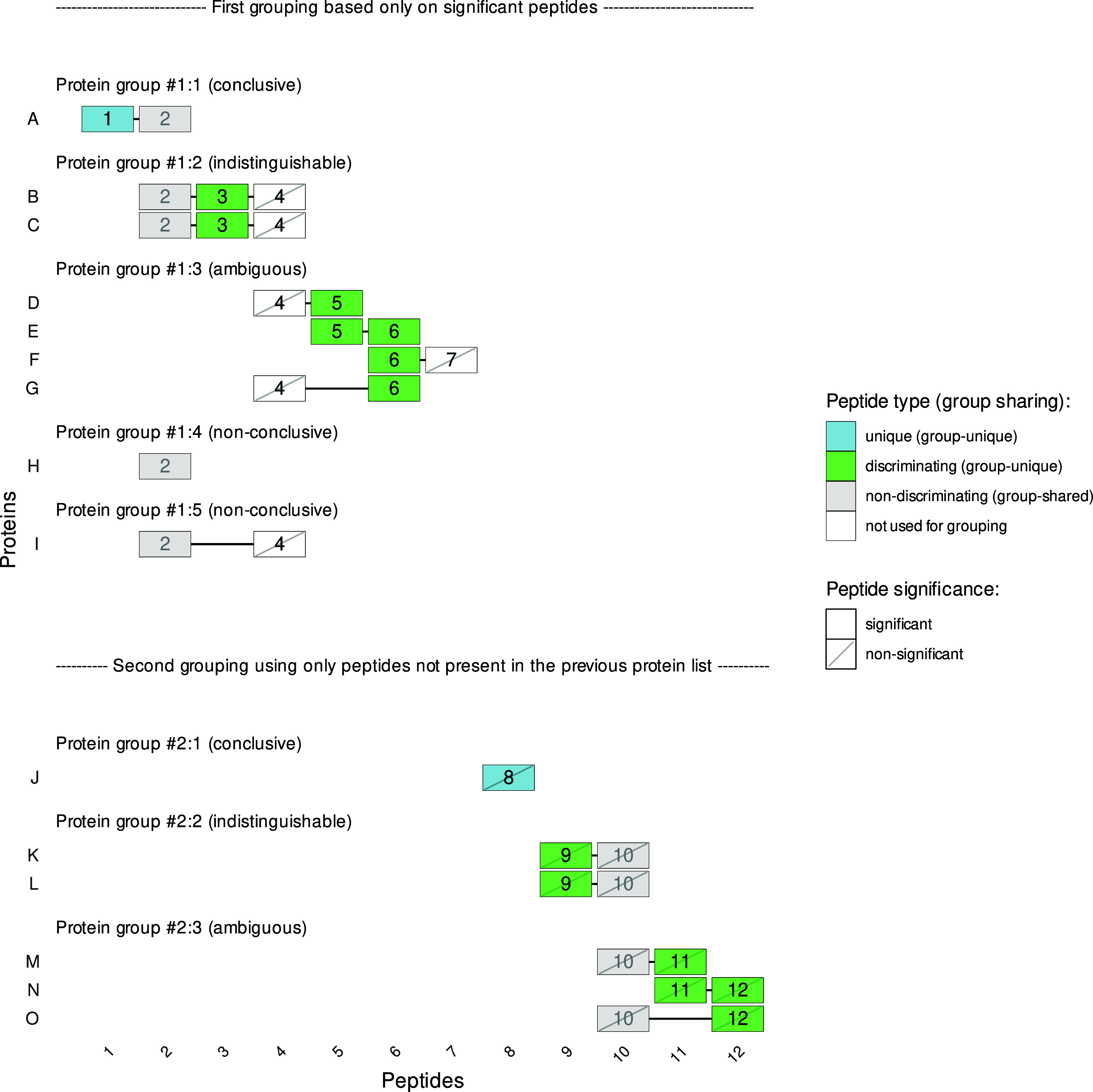
Extended protein groups example from PAnalyzer^6^ to consider non-significant peptides. Proteins are arranged into protein ambiguity groups (PAGs) based on their shared peptides. Unique (protein-unique) and discriminating peptides are group-unique peptides and are used for computing protein-group-level LPGF score, while non-discriminating peptides are shared between different protein groups and do not provide conclusive evidence. Proteins with only non-discriminating peptides are reported as non-conclusive “groups” and discarded from protein-group-level LPGF score computation. Peptides not passing the peptide-level FDR threshold (i.e., peptides 4, 7, and 8..12) are considered non-significant and are not used for arranging protein groups in a first PAnalyzer run, which only considers significant peptides. In contrast to non-discriminating peptides, non-significant peptides are kept and used for protein-group-level LPGF score computation if not shared between groups (e.g., peptide 7). Remaining non-significant peptides (i.e., peptides 8..12) not present in the first PAnalyzer run are used in a second run for building additional protein groups (negative group ID).

LPGF^5^ is a probabilistic protein scoring method designed to compute well-calibrated protein probabilities from peptide identifications. This method models the probability of protein presence by calculating the probability of getting a decoy protein with an equal or lower *p*-value product from *m* identified peptides (e.g., peptides passing a peptide-level FDR threshold) that are selected among *n* matched peptides. This approach results in well-calibrated protein probabilities, where decoy proteins follow the expected uniform distribution, and allows accurate control of false discovery rates at the protein level while increasing identification sensitivity compared to traditional scoring methods. However, the original LPGF method uses protein-unique peptides to compute the protein-level probabilities. In this study, we extend LPGF to handle protein groups, considering only group-unique peptides, thereby preserving informative evidence while allowing for probability estimation at the group level.

Refined FDR (FDRr)^5^ can be viewed as a refinement of the picked FDR (FDRp)^18^ method, avoiding the loss of a certain proportion of proteins that are discarded by FDRp. Since FDRr requires the formation of target-decoy protein pairs, as in FDRp, to extend it to protein groups, we built pairs of protein groups based on the master protein of each group. To select this master protein, proteins within a group are ordered by the number of discriminating peptides, then by the total number of peptides, and finally alphabetically by protein names; the first protein in the list is designated as the master protein.

The b10prot R package has been developed and made available at https://akrogp.github.io/b10prot/ to facilitate the use of the proposed algorithms by the community. The original protein-grouping algorithm of PAnalyzer^6^ is accessible through the panalyzer­() function. The protein grouping approach, considering a peptide-level FDR threshold, as presented in this paper, is implemented in the iwf_grouping function, which internally calls panalyzer twice. Its output can be used as an input to the iwf_pep2group­() function, which also assigns a master protein to each group. LPG scores^5^ can be computed at any level (protein, gene, or group) by using the lpg­() function. Classical decoy/target FDR (FDRn) can be computed directly using the target_decoy_approach function, while the other FDR approaches (FDRp, FDRr, and FDRn) can be computed using the refined_fdr function, which builds target-decoy pairs. The b10prot R package includes a detailed vignette covering the different steps and alternative approaches in the workflows presented.

## Results and Discussion

### Derivation of a Group-Level LPGF Score

The basic idea is to extend the original LPGF^5^ score computation to protein groups by using group-unique peptides, i.e., peptides shared only between proteins within the same protein ambiguity group (PAG). As long as each peptide is assigned to only one group, the same assumptions made for the original LPGF score still hold. To illustrate this, Figure 1 depicts an extended protein grouping example from PAnalyzer^6^ showing different peptide types. Unique (i.e., protein-unique) peptides and discriminating peptides are considered group-unique peptides and are used for computing a protein-group-level LPGF score. In contrast, non-discriminating peptides are shared between different protein groups and are thus discarded in LPGF calculation. Proteins with only non-discriminating peptides are reported as non-conclusive “groups” and are also excluded from protein-group-level LPGF computation.

Non-significant peptides, i.e., peptides not passing the peptide-level identification criterion (e.g., 1% peptide-level FDR), should not be used for arranging the protein groups, but are required by the LPGF model to correctly compute the protein probability, that is, to maintain a uniform protein-group-level decoy score distribution. This is because LPGF takes into account the total number of peptides that uniquely match a given protein or protein group to control random matching. Our proposal to fulfill these two conditions consists of applying the protein-grouping algorithm twice: a first grouping based only on significant peptides, but keeping non-significant ones (e.g., peptides 4 and 7 in Figure 1) to be used for LPGF computation if not shared between groups (e.g., peptide 7); and a second grouping using only peptides not present in the previous protein list (i.e., peptides 8–12 in Figure 1). In this way, non-significant peptides do not affect the protein groups constructed from significant peptides but are kept as required by LPGF. The resulting two sets of protein groups will not share peptides between them, so they can be merged into a single set of protein groups containing all peptides (i.e., both significant and non-significant peptides). Our implementation uses negative group IDs for the second set of protein groups for convenience.

Although we have focused on protein groups, the same assumptions apply to gene groups as well. We will continue to use the term “protein group,” but present the results for both protein groups and gene groups.

Figure 2 shows the proposed protein-group-level LPGF score distribution for the case of decoys. Three tissues from the HPM were tested to assess replicability. Additionally, the results obtained by the classical best-peptide approach have been included for comparison. This best-peptide approach was tested using, as a protein score, the cologarithm of the probability of the best peptide from each protein group (LPM), as previously published.^5^ In contrast to LPM, when LPGF was used as a protein-group-level score, it closely followed the uniform distribution, and this result was reproduced in all data sets analyzed. Thus, the proposed protein-group-level LPGF score acts as a valid probability score.

**Figure 2:**
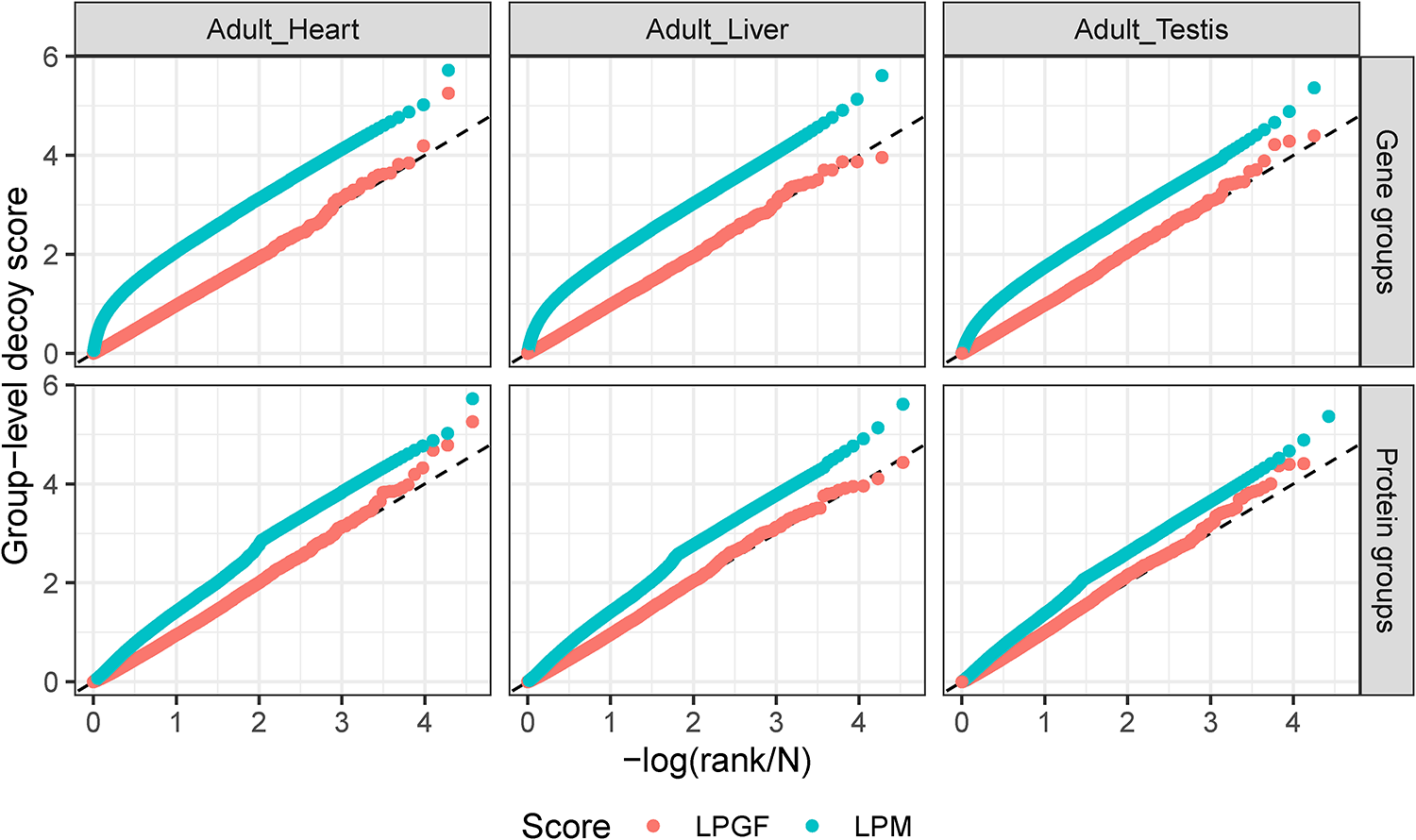
Distribution of different group-level decoy scores using three tissues from the Human Proteome Map (HPM) as separate test data sets. The *y*-axis represents the cologarithm of probabilities, as described in the original LPGF paper,^5^ but now considering group-level unique peptides instead of gene-level or protein-level unique peptides. The *x*-axis represents the cologarithm of the expected uniform protein probabilities. Deviations from the identity line (shown as a dashed black line) indicate that the calculated probabilities are inaccurate. In the case of LPM, deviations are expected since the probability of the best score is not a true measure of protein probability, while LPGF closely follows the uniform distribution, acting as a valid probability score. The upper panels show results for gene groups, and the bottom panel shows results for protein groups.

### Group-Level LPGF Performance

To evaluate the performance of the proposed model, different approaches were considered. Regarding protein inference, we distinguished between using only unique peptides or forming protein groups with discriminating peptides as explained above. Regarding scoring, we evaluated LPGF and the classical best-peptide score expressed as LPM for convenience. Therefore, “unique with LPGF” corresponds to the original LPGF method,^5^ “grouping with LPGF” is the method proposed in this study, while “grouping with LPM” uses the same grouping but selects the best group-unique peptide score as the group score, and thus serves as a reference to assess the performance gain of LPGF at the protein-group level.

Figure 3 shows the identification performance of the different protein inference and scoring approaches mentioned using three tissues from the Human Proteome Map as separate test data sets. The upper panels show results for gene groups, whereas the lower panels show results for protein groups. Full identification ranges have been used to accommodate the large variability observed at the protein level, and also zoomed insets with a fixed range of 500 identifications have been used to better differentiate the remaining cases. As expected, the use of groups increased the number of identifications. In addition, the figure shows that the group-based version of LPGF consistently increases the number of identifications compared to the classical best-peptide approach applied at the group level. Notably, when including isoforms, the use of groups is essential at the protein level to prevent loss of identifications due to shared peptides between isoforms, a phenomenon that is much less pronounced at the gene level and explains why our previous study^5^ reported results only at the gene level. Figure S2 shows the identification performance of the remaining scoring approaches considered in this study. Their behavior is consistent with previous observations; therefore, for simplicity, we focus only on LPM and LPGF in the subsequent analysis.

**Figure 3:**
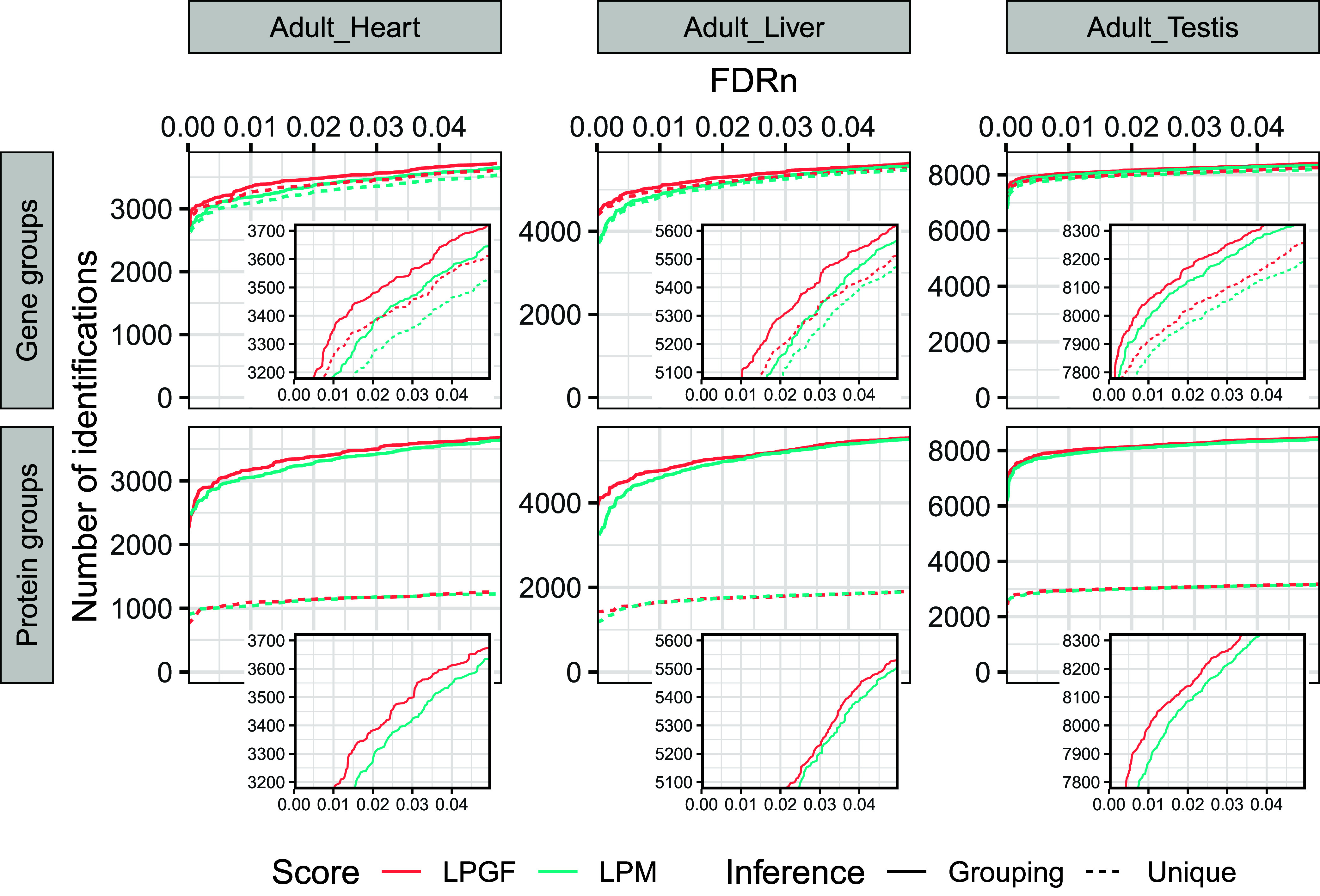
Identification performance of different protein inference and scoring approaches using three tissues from the Human Proteome Map (HPM) as separate test data sets. The *unique* inference approach corresponds to using only gene-level or protein-level unique peptides, while the *grouping* approach uses group-level unique peptides. LPM corresponds to the classical best-peptide approach, but expressed as a cologarithm of probability of the best peptide for convenience, while LPGF is our method now extended to protein or gene groups. The upper panels show results for gene groups, and the bottom panel shows results for protein groups. FDRn is the classical decoy/target FDR estimation following the nomenclature of our previous study.^5^ Insets are included in each panel to provide a zoomed view highlighting the differences between the scoring approaches, each covering a range of 500 identifications. Results were obtained from PSM-level XCorr values following searches performed with Tide.

Table 1 presents the number of identifications at 1% FDR to provide a quantitative comparison. Using gene groups instead of only gene-level unique peptides results in a 2.3% mean identification increase with LPGF and 1.9% with LPM. Notably, at the protein level, it is mandatory to use protein groups because of the inclusion of isoforms in the FASTA database, resulting in an increase in identifications of more than 180% compared to using only protein-level unique peptides. Using LPGF instead of the classical best-peptide approach results in a 3.3% mean increase when using gene groups and a 3.0% increase when using only gene-level unique peptides. The results are similar at the protein-group level, with a 3.0% mean increase when using protein groups and a 2.1% increase when using only protein-level unique peptides.

**Table 1:** Identification Counts and Relative Percentage Improvements at 1% FDR across Three Human Proteome Map (HPM) Tissues Using Different Grouping Levels (Gene or Protein), Protein Inference Strategies (“Group.” or “Uniq.”), and Scoring Approaches (LPGF and LPM)^a^

	Tissue	Group.		Uniq.		LPGF vs LPM		Group. vs Uniq.	
Grouping Level		LPGF	LPM	LPGF	LPM	Group.	Uniq.	LPGF	LPM
Gene	Adult_Heart	3350	3182	3248	3088	5.3%	5.2%	3.1%	3.0%
	Adult_Liver	5061	4869	4962	4815	3.9%	3.1%	2.0%	1.1%
	Adult_Testis	8055	7991	7912	7854	0.8%	0.7%	1.8%	1.7%
					Mean	3.3%	3.0%	2.3%	1.9%
Protein	Adult_Heart	3171	3054	1092	1031	3.8%	5.9%	190%	196%
	Adult_Liver	4760	4588	1648	1657	3.7%	–0.5%	189%	177%
	Adult_Testis	7991	7866	2954	2926	1.6%	1.0%	171%	169%
					Mean	3.0%	2.1%	183%	181%

a “Group.” denotes identifications obtained using peptides that are unique at the group level (i.e., peptides mapping uniquely to a protein group or gene group), whereas “Uniq.” denotes identifications obtained using peptides that are unique at the individual protein or gene level (i.e., without considering grouping). LPGF is the scoring method extended here to protein and gene groups, while LPM corresponds to the classical best-peptide approach. Columns “LPGF vs LPM” report the relative increase in identifications obtained with LPGF compared to LPM, while “Group. vs Uniq.” reports the relative increase obtained when using group-level unique peptides (“Group.”) instead of individual-level unique peptides (“Uniq.”). Results were obtained starting from PSM-level XCorr scores following database searches performed with Tide.

These results were obtained starting from PSM-level XCorr values following searches performed with Tide. Figure S1 and Table S1 show the results when starting from peptide-level scores computed by Percolator after the Tide searches. This improved separation of targets and decoys reduces the impact of LPGF at higher levels, although the overall behavior remains similar. In this case, using LPGF instead of the classical best-peptide approach results in a 1.3% mean increase when using gene groups and a 1.1% increase when using only gene-level unique peptides. The results are similar at the protein-group level, with a 1.6% mean increase when using protein groups and a 1.2% increase when using only protein-level unique peptides. The use of peptide property prediction tools such as Prosit,^19^ MSBooster,^20^ and pDeep^21^ could further improve target-decoy separation and reduce the relative impact of LPGF at higher levels. Integrating such features within the proposed framework remains an interesting direction for future work.

### Accuracy of Group-Level LPGF Using a Gold Standard

To evaluate the accuracy of the proposed protein-group-level LPGF method, we selected a gold-standard protein inference data set^8^ with known protein content. This data set consists of 191 overlapping pairs of PrEST (Protein Epitope Signature Tag) sequences from the Human Proteome Atlas project.^22^ These protein pairs share at least one identical fully tryptic peptide. Each protein was randomly assigned to one of the pools (A or B) that were created. A third pool was created by mixing pools A and B, resulting in a pool A+B. To calculate an entrapment FDR,^23^ the study also included a set of entrapment sequences consisting of 1000 representative PrEST fragments that were not selected for the mixtures and hence were absent from the samples.

Figure 4 shows the accuracy of the tested protein inference and scoring approaches mentioned using these PrEST gold-standard data sets. The entrapment FDR was normalized by the prior probability of the PrEST being absent, the so-called π_*A*_.^24^ In the original study,^8^ the only inference approach that handled in a satisfying manner the mixtures with shared peptides between present and absent proteins (mixtures A and B) was using only protein-level unique peptides. Our results show that both the grouping inference approach and the protein-group-level LPGF score performed well across the different data sets.

**Figure 4:**
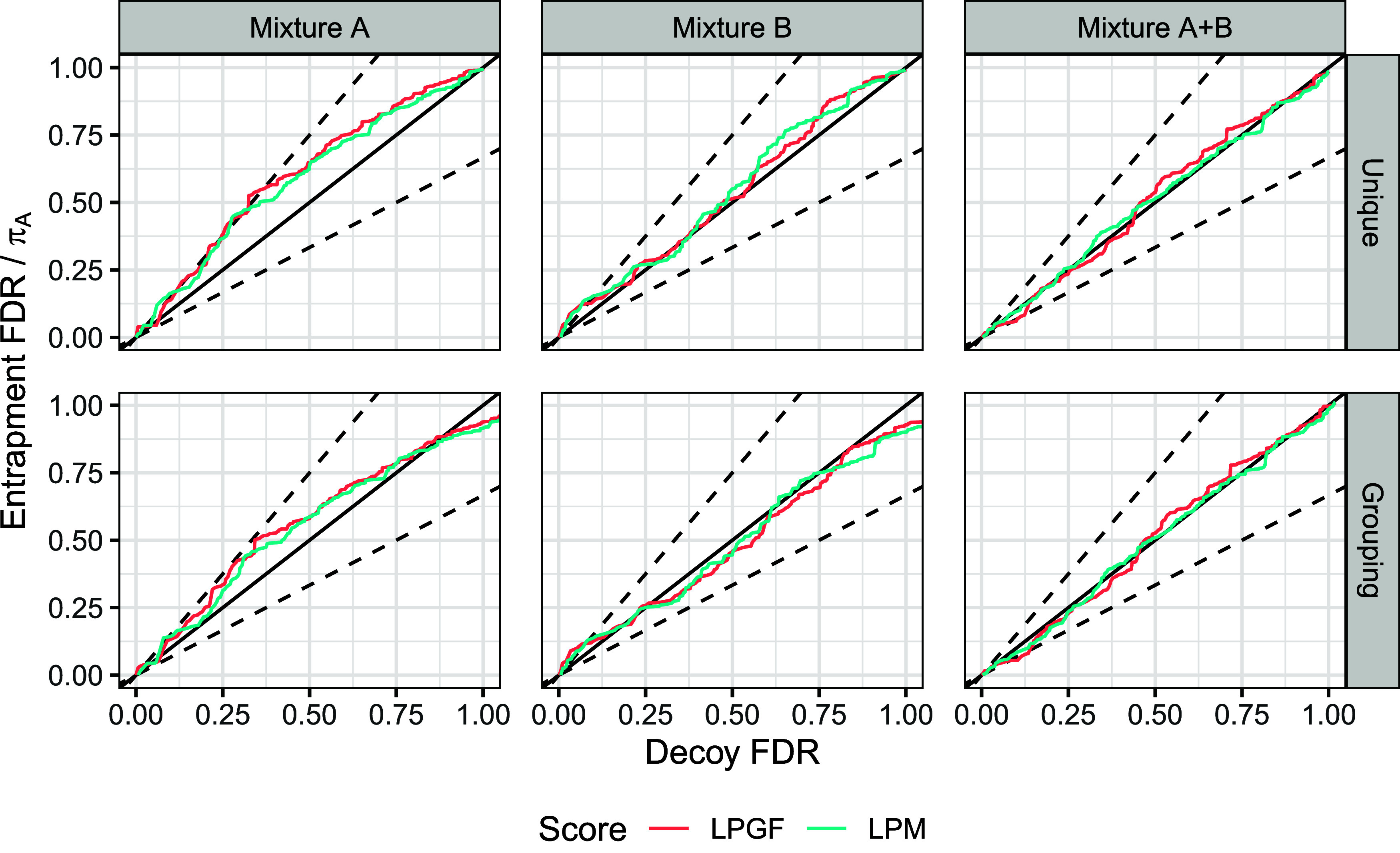
Accuracy of the tested protein inference and scoring approaches using the PrEST gold-standard data sets. The entrapment FDR was normalized by the prior probability of the PrEST being absent, the so-called π_*A*_.^24^ Mixtures A and B share peptide sequences with absent proteins, while mixture AB shares peptide sequences between present proteins. The *unique* inference approach corresponds to using only protein-level unique peptides, while the *grouping* approach uses protein-group-level unique peptides. LPM corresponds to the classical best-peptide approach, but expressed as a cologarithm of probability for convenience, while LPGF is our score now extended to protein groups. The region between *y* = *x*/1.5 and *y* = 1.5 · *x* (dashed lines) was deemed well-calibrated.

### FDR Evaluation Using an Entrapment Database

The results on the PrEST data set provide a good benchmark for FDR calibration, but another best practice is to use entrapment databases to evaluate FDR control on realistically sized data sets. We used FDRBench^15^ to include entrapment proteins in the human proteome FASTA file and to estimate the false discovery proportion (FDP) after repeating the analysis at the protein-group level, starting from PSM-level XCorr values obtained from Tide searches.

For this extended analysis, we decided to include both the classical decoy/target FDR estimation approach (FDRn) and the refined FDR (FDRr) we previously described.^5^ FDRr can be viewed as a refinement of the picked FDR^18^ (FDRp) method, avoiding the loss of a certain proportion of proteins that are discarded by the FDRp. Since FDRr requires the formation of target-decoy protein pairs, as in FDRp, to extend it to protein groups, we built pairs of protein groups based on the master protein of each group.

Figure 5 shows the entrapment evaluation results of FDR control across the three tissues of the Human Proteome Map (HPM) using LPGF as the protein-group-level score and the different FDR estimations. The upper panels show that protein-group-level LPGF with FDRn approaches from below the so-called combined method,^15^ which provides an estimated upper bound of the FDP, suggesting successful FDR control. The lower panels show that using FDRr, the results approach from below the so-called paired method,^15^ which provides a more accurate estimation of the FDP.

**Figure 5:**
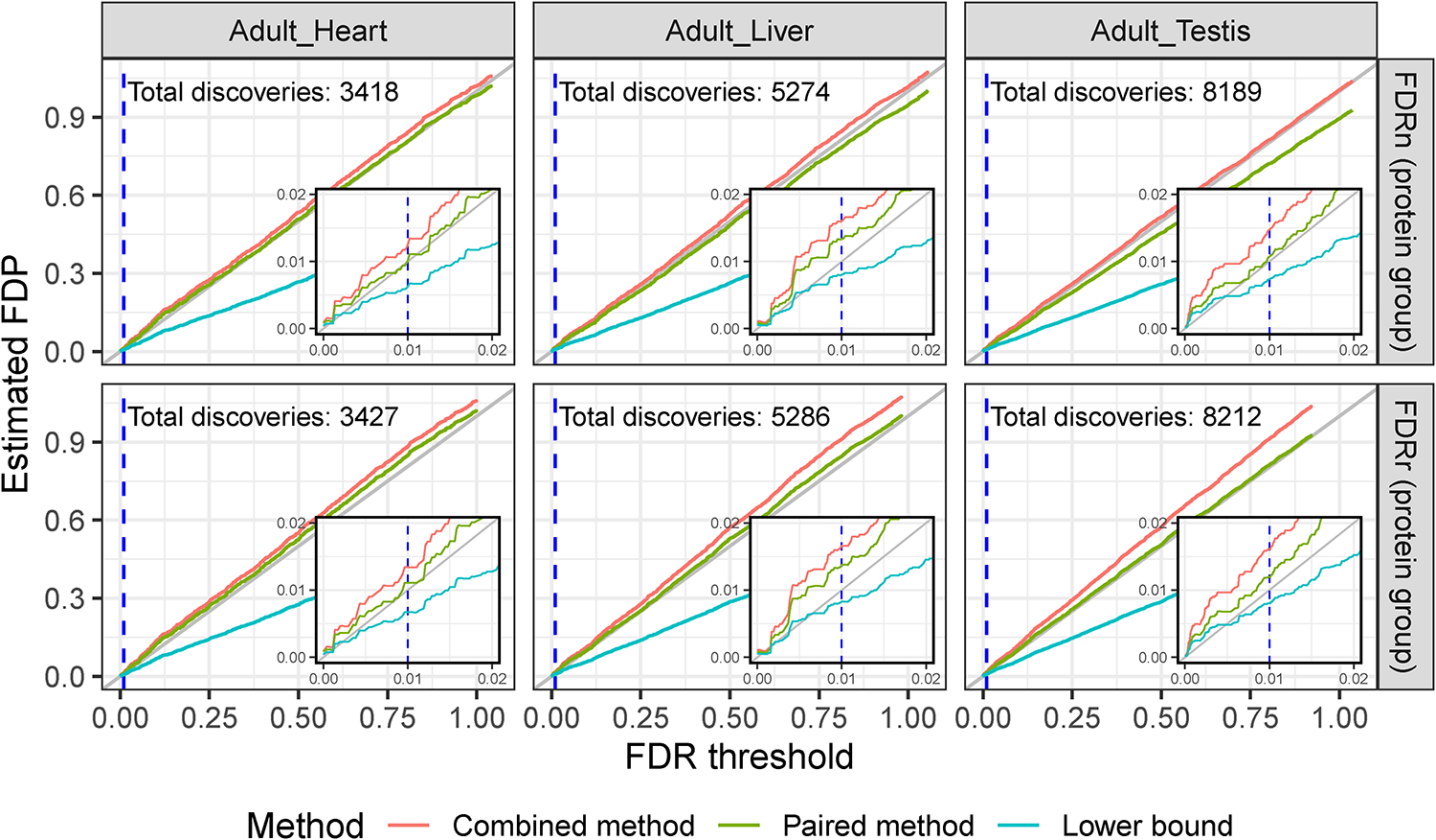
Entrapment evaluation of the FDR control across three tissues of the Human Proteome Map (HPM) using LPGF as the protein-group-level score and different FDR estimations. FDRn is the classical decoy/target estimation, while FDRr is our original refined estimation,^5^ now extended to protein groups. Insets show a zoomed view of the 0–2% FDR region.

## Conclusions

In this study, we present an extension of our previously published LPGF protein-level probabilistic model to protein groups, effectively addressing two key challenges in protein identification with shotgun proteomics: the ambiguity introduced by shared peptides and the control of false discovery rates at the protein level. By leveraging PAnalyzer to define protein groups and identify group-unique peptides, our approach preserves informative evidence that would otherwise be discarded when only protein-unique peptides are considered. The original PAnalyzer grouping algorithm has been maintained, and we further introduce a two-round grouping strategy: the first round forms groups using significant peptides, while a second round incorporates non-significant peptides, which are also required for proper computation of LPGF probabilities.

The group-level LPGF score provides well-calibrated protein probabilities across multiple tissues from the Human Proteome Map. Compared to the classical best peptide scoring approach (LPM), LPGF consistently improves identification rates, with an approximate increase of 3% when using groups and 2% when using only unique peptides. Moreover, forming groups increases the number of identifications by an average of 2% with LPGF, highlighting the benefits of explicitly modeling peptide sharing.

Using a PrEST-based gold-standard data set, we demonstrate that the group-level LPGF maintains accurate control of false discovery rates and performs reliably even when peptides are shared between present and absent proteins. An additional entrapment database analysis confirms that LPGF provides reliable FDR control at the protein-group level for realistically sized data sets. Our refined FDR (FDRr) provides a closer approximation to the paired method for FDP estimation, while the classical FDR (FDRn) is more conservative, being similar to the combined method for the FDP estimation. These results indicate that the proposed method combines the advantages of protein grouping with rigorous probabilistic scoring, enabling both increased sensitivity and robust error control. Extending this evaluation to very large-scale data sets comprising tens or hundreds of samples, where FDR control becomes increasingly challenging, would be an interesting direction for future work.

Finally, to facilitate adoption by the community, we provide the open-source *R* package b10prot, which integrates protein grouping via PAnalyzer with protein- and group-level LPGF score computation and FDRr estimation. This package allows researchers to apply our methodology in a reproducible and user-friendly manner, bridging the gap between theoretical probabilistic scoring and practical, large-scale proteomics analyses.

## Supplementary Material



## Data Availability

All data sets, scripts, and results required to reproduce the analyses and figures presented in this study are publicly available on Zenodo under DOI 10.5281/zenodo.19115195 (10.5281/zenodo.19115195). The analyses are based in part on previously published data sets available via the ProteomeXchange consortium through the PRIDE database: the HPM data set (accession PXD000561) and the PrEST data set (accession PXD008425), which can be accessed at https://www.ebi.ac.uk/pride/archive/. The Zenodo repository includes the FASTA databases used (with and without entrapment proteins), the search results obtained with Tide and Tide+Percolator, as well as the processed result tables and the *R* notebooks used to generate the reported analyses and figures.

## Notes

Identification counts and relative percentage improvements of different protein inference and scoring approaches using peptide-level scores computed by Percolator (Table S1); identification performance of different protein inference and scoring approaches using peptide-level scores computed by Percolator (Figure S1); and extended identification performance evaluations using alternative scoring methods (Figure S2) (PDF)
