# Supplementary material for: Improved Protein Identification in Shotgun Proteomics with a Group-Level Extension of the LPGF Model
Source: J Proteome Res. 2026 Jul 20;25(8):3937–45. doi: 10.1021/acs.jproteome.5c01253 (PMC13459553; doi:10.1021/acs.jproteome.5c01253)
Supplement: Supplementary file 1 [file pr5c01253_si_001.pdf]

SUPPLEMENTARY MATERIAL TO

Improved Protein Identification in Shotgun Proteomics with a Group-Level Extension of the LPGF Model

Gorka Prieto*[1] and Jesús Vázquez[2,3]

[1]Department of Communications Engineering, University of the Basque Country (UPV/EHU), 48013 Bilbao, Spain
[2]Cardiovascular Proteomics Lab, Centro Nacional de Investigaciones Cardiovasculares Carlos III (CNIC), 28029 Madrid, Spain
[3]CIBER de Enfermedades Cardiovasculares (CIBERCV), 28029 Madrid, Spain

*Email: gorka.prieto@ehu.eus

List of Figures

Figure S1 Identification performance of different protein inference and scoring approaches using peptide-level scores computed by Percolator. S2
Figure S2 Extended identification performace evaluations using alternative scoring methods. . . . S3

List of Tables

Table S1 Identification counts and relative percentage improvements of different protein inference and scoring approaches using peptide-level scores computed by Percolator. S4

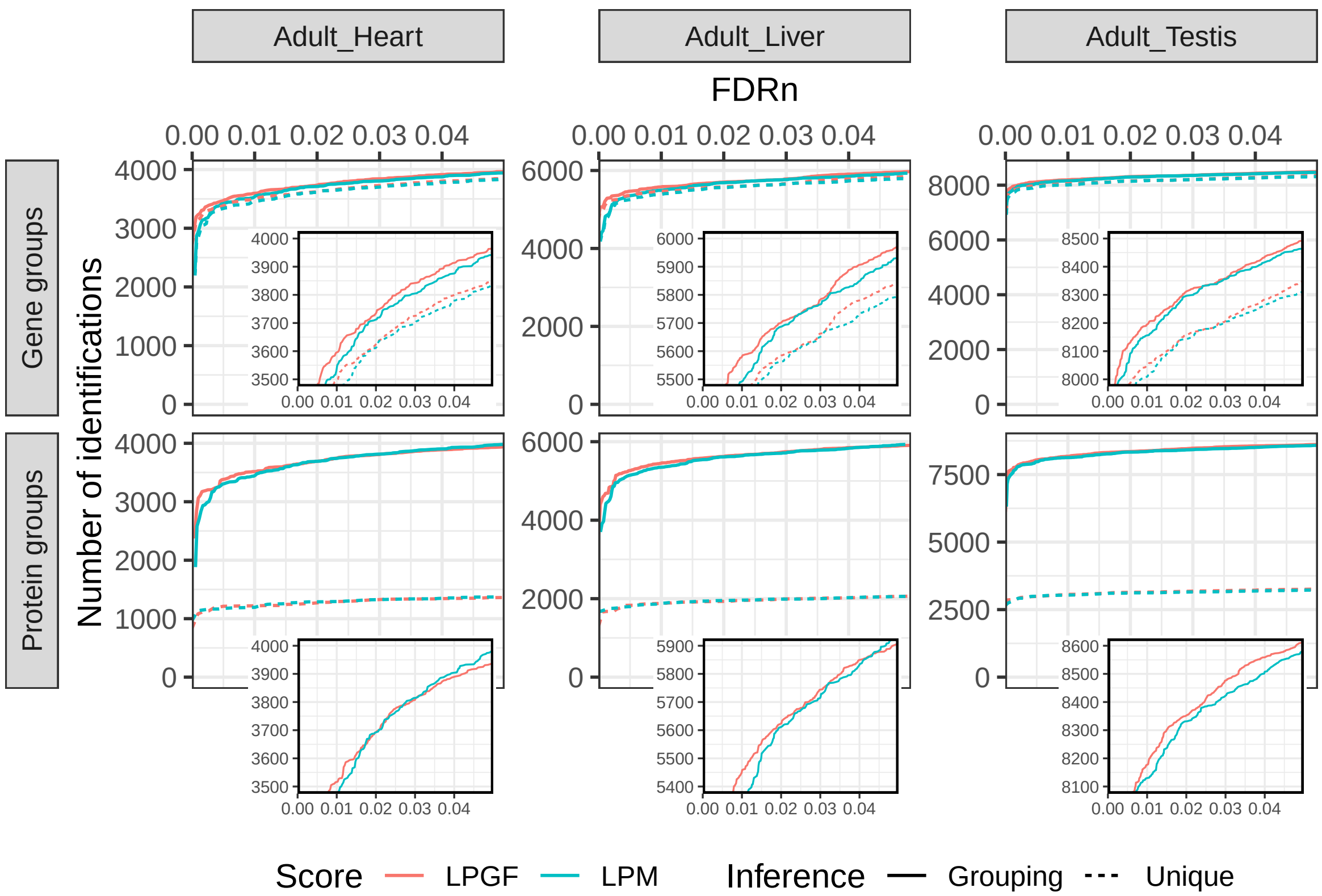

Figure S1: Identification performance of different protein inference and scoring approaches using three tissues from the Human Proteome Map (HPM) as separate test datasets. The *unique* inference approach corresponds to using only gene-level or protein-level unique peptides, while the *grouping* approach uses group-level unique peptides. LPM corresponds to the classical best-peptide approach, but expressed as a cologarithm of probability of the best peptide for convenience, while LPGF is our method now extended to protein or gene groups. The upper panels show results for gene groups, and the bottom panel shows results for protein groups. FDRn is the classical decoy/target FDR estimation following the nomenclature of our previous study. Insets are included in each panel to provide a zoomed view highlighting the differences between the scoring approaches, each covering a range of 500 identifications. Results were obtained from peptide-level scores computed by Percolator following Tide searches.

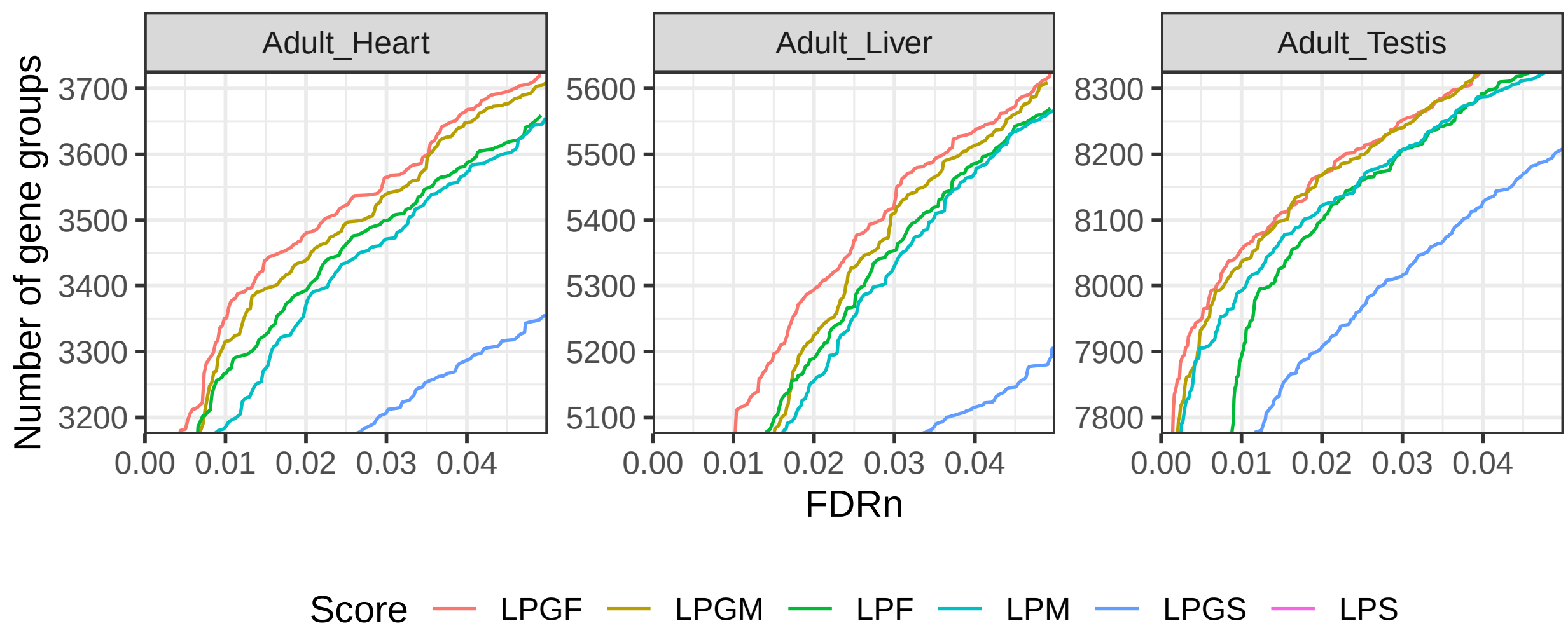

Figure S2: Gene group-level identification performance of different scoring approaches using three tissues from the Human Proteome Map (HPM) as separate test datasets. FDRn is the classical decoy/target FDR estimation following the nomenclature of our previous study. Results were obtained from PSM-level XCorr values following searches performed with Tide.

Table S1: Identification counts and relative percentage improvements at 1% FDR across three Human Proteome Map (HPM) tissues using different grouping levels (gene or protein), protein inference strategies ("Group." or "Uniq."), and scoring approaches (LPGF and LPM). "Group." denotes identifications obtained using peptides that are unique at the group level (i.e., peptides mapping uniquely to a protein group or gene group), whereas "Uniq." denotes identifications obtained using peptides that are unique at the individual protein or gene level (i.e., without considering grouping). LPGF is the scoring method extended here to protein and gene groups, while LPM corresponds to the classical best-peptide approach. Columns "LPGF vs LPM" report the relative increase in identifications obtained with LPGF compared to LPM, while "Group. vs Uniq." reports the relative increase obtained when using group-level unique peptides ("Group.") instead of individual-level unique peptides ("Uniq."). Results were obtained from peptide-level scores computed by Percolator following Tide searches.

Grouping level	Tissue	Group.		Uniq.		LPGF vs LPM		Group. vs Uniq.	
		LPGF	LPM	LPGF	LPM	Group.	Uniq.	LPGF	LPM
Gene	Adult_Heart	3593	3531	3494	3452	1.8%	1.2%	2.8%	2.3%
	Adult_Liver	5577	5491	5467	5386	1.6%	1.5%	2.0%	1.9%
	Adult_Testis	8194	8156	8047	8009	0.5%	0.5%	1.8%	1.8%
					Mean	1.3%	1.1%	2.2%	2.0%
Protein	Adult_Heart	3516	3439	1220	1187	2.2%	2.8%	188%	190%
	Adult_Liver	5448	5346	1876	1882	1.9%	-0.3%	190%	184%
	Adult_Testis	8176	8130	3059	3030	0.6%	1.0%	167%	168%
					Mean	1.6%	1.2%	182%	181%
